# Association between offensive behaviors and burnout and depression risks in health workers *

**DOI:** 10.1590/1518-8345.6683.3987

**Published:** 2023-09-18

**Authors:** Luiza Salvador Rohwedder, Fabio Leandro da Silva, Bianca Biason Albuquerque, Rosângela Sousa, Tatiana de Oliveira Sato, Vivian Aline Mininel

**Affiliations:** 1 Universidade Federal de São Carlos, Departamento de Enfermagem, São Carlos, SP, Brasil.; 2 Becaria de la da Fundação de Amparo à Pesquisa do Estado de São Paulo.; 3 Universidade Federal de São Carlos, São Carlos, SP, Brasil.; 4 Becaria del Conselho Nacional de Desenvolvimento Científico e Tecnológico.

**Keywords:** Workplace Violence, Depression, Professional Burnout, Occupational Health, Working Conditions, Occupational Risks, Violencia Laboral, Depresión, Agotamiento Profesional, Salud Laboral, Condiciones de Trabajo, Riesgos Laborales, Violência no Trabalho, Depressão, Esgotamento Profissional, Saúde do Trabalhador, Condições de Trabalho, Riscos Ocupacionais

## Abstract

**Objective:**

to evaluate the occurrence of offensive behaviors at work, their characteristics and association with sex, stress, burnout and depression in health workers.

**Method:**

a cross-sectional, descriptive and quantitative study carried out with 125 workers from the Brazilian Unified Health System. The data were collected from June 2021 to April 2022 through three self-applied questionnaires that assess personal and occupational characteristics; offensive behaviors, stress and burnout; and depressive symptoms. Descriptive statistics, the chi-square association test and logistic regression analysis were applied.

**Results:**

44% of the sample reported 83 behaviors, with threats of violence as the most frequent ones (26%). Nursing technicians/assistants, nurses and physicians were the most exposed professionals. The main aggressors were the patients, except for bullying, which was perpetrated by co-workers (48%). There was an association between offensive behaviors and burnout (OR: 4.73; 95% CI: 1.29-17.3; p=0.02) and between offensive behaviors and depression symptoms (OR: 1.05; 95% CI: 1.01-1.10; p=0.02).

**Conclusion:**

the occurrence of offensive behaviors in health work is frequent and characteristic and burnout and depressive symptoms respectively increased 4.73 and 1.05 times the chances of workers suffering these offensive behaviors in the work environment.

## Abstract (es)

**Objetivo:**

evaluar la incidencia de conductas ofensivas en el trabajo, las características y la asociación con el sexo, el estrés, el burnout y la depresión en trabajadores de la salud.

**Método:**

estudio transversal, descriptivo, cuantitativo, realizado con 125 trabajadores del Sistema Único de Salud brasileño. Los datos fueron recolectados entre junio de 2021 y abril de 2022, por medio de tres cuestionarios autoadministrados que evalúan características personales y ocupacionales; comportamientos ofensivos, estrés y burnout y síntomas de depresión. Se aplicó estadística descriptiva, prueba de asociación chi-cuadrado y análisis de regresión logística

**Resultados:**

el 44% de la muestra declararon 83 conductas y las amenazas de violencia fueron las más frecuentes (26%). Técnicos/auxiliares de enfermería, enfermeros y médicos fueron los profesionales más expuestos. El principal agresor fue el paciente; excepto en el caso del bullying, que fue perpetrado por los compañeros de trabajo (48%). Hubo asociación entre conductas ofensivas y burnout (OR: 4,73; IC 95%: 1,29-17,3; p=0,02) y entre conductas ofensivas y síntomas de depresión (OR: 1,05; IC 95%: 1,01-1,10; p=0,02).

**Conclusión:**

la práctica de conductas ofensivas en el trabajo en salud es frecuente y característica; el burnout y los síntomas de depresión aumentaron, respectivamente, 4,73 y 1,05 veces las posibilidades de que el trabajador sufriera esas conductas ofensivas en el ambiente de trabajo.

## Abstract (pt)

**Objetivo:**

avaliar a ocorrência de comportamentos ofensivos no trabalho, suas características e associação com o sexo, estresse, burnout e depressão em trabalhadores de saúde.

**Método:**

estudo transversal, descritivo, de natureza quantitativa, realizado com 125 trabalhadores do Sistema Único de Saúde brasileiro. Os dados foram coletados no período de junho de 2021 a abril de 2022, por meio de três questionários autoaplicáveis que avaliam as características pessoais e ocupacionais; comportamentos ofensivos, estresse e burnout e sintomas depressivos. Estatística descritiva, teste de associação Qui-Quadrado e análise de regressão logística foram aplicados.

**Resultados:**

44% da amostra referiram 83 comportamentos, sendo as ameaças de violência mais frequentes (26%). Técnicos/auxiliares de enfermagem, enfermeiros e médicos foram os profissionais mais expostos. O principal agressor foi o paciente, exceto quanto ao bullying, perpetrado por colegas de trabalho (48%). Houve associação entre comportamentos ofensivos e burnout (OR: 4,73; IC 95%: 1,29-17,3; p=0,02) e entre comportamentos ofensivos e sintomas de depressão (OR: 1,05; IC 95%: 1,01-1,10; p=0,02).

**Conclusão:**

a ocorrência de comportamentos ofensivos no trabalho em saúde é frequente e característica; o burnout e os sintomas depressivos aumentaram, respectivamente, 4,73 e 1,05 vezes as chances de o trabalhador sofrer estes comportamentos ofensivos no ambiente de trabalho.

**Highlights:**

**(1)** Threats of violence represent the most frequent type of offensive behavior

**(2)** Nursing professionals and physicians are more affected by violence at work

**(3)** Victims of violence at work are more likely to have depressive symptoms

**(4)** Having suffered violence at work increases by almost five times the chances of burnout

## Introduction

Health workers are exposed to various risks in their daily work, such as psychosocial, biological and organizational ones ^(^ 1 ^)^. This group is also more exposed to adverse social behaviors ^(^ 2 ^)^, with violence at work as a highly incident phenomenon ^(^ 3 ^-^ 4 ^)^ that has been increasing over the years ^(^ 7 ^)^, especially in the first two years of the COVID-19 pandemic, which exerted pressure on health systems and, consequently, on the workers linked to them ^(^ 8 ^)^. 

The International Labor Organization (ILO) defines violence and harassment at work as a set of threats or unacceptable behaviors and practices that result or may result in physical, psychological, sexual or economic harms ^(^ 9 ^)^. According to the framework adopted ^(^ 10 ^)^, the term “work-related violence” can be defined as the presence of offensive behaviors in the work environment, including unwanted sexual attention, threats of violence, physical violence and bullying. 

Unwanted sexual attention is one of the sexual harassment dimensions and includes verbal and non-verbal behaviors characterized as offensive, unwanted, non-reciprocal and of a sexual nature ^(^ 11 ^)^, which can convey an implicitly coercive message when practiced by a boss/supervisor who has the power to hire, dismiss and promote a professional career. 

Threats of violence include promises to use physical force or power and result in fear, sexual harms or other negative consequences for the victim ^(^ 12 ^)^. Physical violence involves the use of physical force and includes acts such as beating, slapping, kicking, stabbing, throwing, pushing, biting and pinching, resulting in actual physical harms ^(^ 13 ^)^. Also known as moral harassment or mobbing, bullying refers to situations of non-sexual harassment, offenses, social exclusion or harms caused intentionally, recurrently and over a given period of time ^(^ 13 ^)^. 

Among the factors that contribute to the occurrence of offensive behaviors in health work, organizational factors stand out, such as restrictive policies, insufficient number of workers, professional inexperience, lack of training ^(^ 14 ^)^ and precarious working conditions that impact on the reduction of care quality. Aspects related to the aggressors, such as the patients’ disease profile and the anxiety or stress of their companions ^(^ 15 ^)^ can favor such behaviors and are frequently pointed out by the workers themselves to justify situations of violence and redeem the aggressor’s guilt ^(^ 6 ^)^. 

Work-related violence has been naturalized in the routine of health services and few measures have been implemented to face it, treat it and prevent it ^(^ 6 ^)^, even though its countless repercussions, which include physical and/or psychological distress and increase the risks for the workers’ mental health ^(^ 16 ^)^. Care quality is also affected by this context, in view of the reduction in the workforce, either due to illness or wear out in the team members ^(^ 17 ^)^. 

Despite the relevance of the subject matter, research studies on this topic are still scarce in the Brazilian scenario ^(^ 6 ^,^ 14 ^)^, especially considering the recent context of the COVID-19 pandemic, in which there was greater emotional and physical effort by health workers and more precarious working conditions ^(^ 18 ^)^, with the need for a closer look at this population segment. 

Early identification of the different forms of violence in the work environment and their consequences offers subsidies to managers in proposing prophylactic and control measures against these events ^(^ 19 ^)^. In this sense, this study aimed at evaluating the occurrence of offensive behaviors at work, their characteristics and association with sex, stress, burnout and depression in health workers. 

## Method

### Study design

A cross-sectional, descriptive and quantitative study based on the recommendations set forth in the Checklist for Reporting Results of Internet E-Surveys (CHERRIES) ^(^ 20 ^)^ guide and derived from the HEROES (HEalth conditions of healthcaRe wOrkErS) longitudinal survey ^(^ 21 ^)^, whose objective was to evaluate psychosocial aspects at work, sleep characteristics, musculoskeletal symptoms and depression in Unified Health System ( *Sistema Único de Saúde* – SUS) health workers. 

### Setting and period

The e-survey covered the national territory with data collected through a free electronic form from Google Forms, aiming to respect the contact restrictions imposed by the COVID-19 pandemic, from June 2021 to April 2022.

### Population and sampling

Recruitment of the potential participants was carried out through Internet channels, through the press, social networks and email addresses available on institutional websites. Two researchers participated in interviews on local radios (two stations) and wrote articles for dissemination, and nine students (five undergraduates and four graduates) produced materials for the profiles of the HEROES project on the Instagram, Facebook and YouTube networks. In addition, email messages were sent to public hospitals, Health Departments and Units and Class Bodies (Nursing, Physiotherapy, Psychology, Nutrition, Medicine) for dissemination.

The inclusion criteria were being a SUS health service worker, aged between 18 and 60 years old and working in care activities. Participation was voluntary and there was no financial incentive. Students, retirees, duplicate answers and inconsistent data were excluded. The convenience sample consisted of 125 individuals.

### Instruments

Three instruments were used: (i) a sociodemographic and work-related questionnaire containing questions associated with gender, age, marital status, schooling, health history, life habits and work history; (ii) the short version of the Copenhagen Psychosocial Questionnaire II ^(^ 22 ^-^ 23 ^)^, validated for the Portuguese language spoken in Brazil (COPSOQ II-Br), with Cronbach’s alpha values between 0.70 and 0.87 ^(^ 22 ^)^ and (iii) the Beck Depression Inventory II, validated for the Portuguese language spoken in Brazil (BDI-II) with a Cronbach’s alpha of 0.93 ^(^ 24 ^)^. The three instruments were incorporated into the Google Forms electronic form and the participants were able to review their responses and change them as they answered the questions. Usability and technical functionality of the electronic form containing the three questionnaires were tested before release. 

COPSOQ II is used to assess the psychosocial aspects at work in different populations, contributing to studies in the Occupational Health area and being an important ally to programs for the prevention of psychosocial risks ^(^ 10 ^,^ 23 ^)^. The short version of COPSOQ II-Br consists of 40 questions, divided into seven domains: 1. Work demands; 2. Work organization and content; 3. Interpersonal relationships; 4. Work-individual interface; 5. Workplace values; 6. Health and well-being and 7. Offensive behaviors ^(^ 22 ^)^. The questions are scored using a five-point Likert scale, with the score calculated according to the number of questions in each domain, on a scale from zero to eight points (ranges from 0 to 3 points, from 0 to 4 points, from 0 to 6 points, and from 0 to 8 points). The values obtained are classified into “favorable situation for health” (green), “intermediate situation” (yellow) and “risk for health” (red) ^(^ 22 ^)^. Dimension 7 - “Offensive behaviors” is classified differently, as it contains dichotomous options, where answering “yes” to at least one type of offensive behavior indicates the presence of violence at work. This research included the burnout and stress dimensions from COPSOQ II-Br, which are part of Domain 6 - “Health and well-being” and of Domain 7 - “Offensive behaviors”, consisting of four dimensions: unwanted sexual attention, threats of violence, physical violence and bullying. 

BDI-II assesses depression symptoms through 21 self-reported questions ^(^ 24 ^)^. For each question, there is a four-point scale from zero to three points: zero means no symptoms and three represents presence of severe symptoms. The participants must respond based on the last two weeks, including the day when the answer is given. If multiple statements describe their condition, they should tick the answer with the highest number. The total score is calculated by adding the results of all 21 questions, varying from zero to 63 points. Interpretation of the results is based on scoring ranges that indicate specific categories of depression, as follows: from zero to 13 points: no depression; from 14 to 19 points: average depression; from 20 to 28: moderate depression and from 29 to 63: severe depression ^(^ 24 ^)^. 

### Data treatment and analysis

Only completely completed questionnaires were analyzed. Descriptive statistical analysis was used for all variables, with the aid of the Statistical Package for the Social Sciences (SPSS) software, version 26.0 and by means of the R language (version 4.1.2).

Due to the qualitative nature of the data, the Chi-square test was applied to test the association between offensive behaviors, sex and risk of stress and burnout at work.

Domain 7 - “Offensive behaviors” from COPSOQ II-Br was considered as an outcome variable (dependent), comprised by four nominal qualitative variables (dichotomous). Answering “yes” to any of the four dimensions already indicates a health risk (red). The independent variables were extracted from COPSOQ II-Br Domain 6 - “Health and well-being”, in the stress and burnout dimensions and categorical qualitative variables from the BDI-II, according to the classification. The binomial logistic regression analysis tested the association between having suffered any type of offensive behavior (dependent variable) and the independent variables of gender, having children (yes or no), stress (health risk classification - red), burnout (health risk classification - red) and depression symptoms (total score obtained - discreet quantitative variable). The significance level adopted was 5%.

### Ethical aspects

The study met the ethical requirements for research involving human beings, as recommended by National Health Council resolutions No. 466/2012 and No. 510/2016, and was approved by the Research Ethics Committee ( *Comitê de Ética em Pesquisa*, CEP) under CAAE No. 39705320.9.0000.5504. All participants consented to the Free and Informed Consent Form (FICF) before starting the questionnaires. 

## Results

A total of 125 health professionals participated in the research, most of them female (83%), with a mean age of 37.5 years old [Standard Deviation (SD)=8.3], self-declared white-skinned (71%), married (57%), without children (52%), with graduate studies (63%), and from the following professional categories: nurses (36.0%), nursing technicians/assistants (22.4%), physical therapists (20.8%), physicians (6.4%), dentists (3.2%) and other professionals (11.2%) (a community health worker, two social workers, two pharmacy assistants, one ambulance driver, one home care coordinator, two speech therapists, two nutritionists, two occupational therapists and a psychologist). The participants came from the Southeast (79.2%), South (11.2%), Northeast (4.8%), Midwest (3.2%) and North (1.6%) regions. Table 1 presents social and demographic characteristics of the participants according to professional categories. 

Most of the professionals worked in hospitals (49%), with a weekly hour load of 40 hours (48%), contractual regime governed by the Consolidation of Labor Laws (48%) and monthly incomes of three to six minimum wages (MWs) (39%). Thirty-one percent stated having more than one employment contract, as presented in Table 2. 

**Table 1 -:** Social and demographic characteristics of health professionals (n=125). Brazil, 2021-2022

Characteristics	Total (n=125)	Dentists (n=4)	Physical Therapists (n=26)	Nurses (n=45)	Nursing technicians/ Assistants (n=28)	Physicians (n=8)	Others (n=14)
Age (years old)	37.5 (8.3) ^*^	28.0 (6.2) ^*^	36.3 (7.3) ^*^	38.2 (7.3) ^*^	39.4 (9.0) ^*^	35.0 (11.3) ^*^	37.4 (9.2) ^*^
Gender							
Female	104 (83.2)	3 (75.0)	22 (84.6)	39 (86.7)	25 (89.3)	3 (37.5)	12 (85.7)
Male	21 (16.8)	1 (25.0)	4 (15.4)	6 (13.3)	3 (10.7)	5 (62.5)	2 (14.3)
Skin color/Race							
White	89 (71.2)	2 (50.0)	20 (76.9)	33 (73.3)	18 (64.3)	6 (75.0)	10 (71.4)
Brown	29 (23.2)	2 (50.0)	5 (19.3)	9 (20.0)	7 (25.0)	2 (25.0)	4 (28.6)
Asian	1 (0.8)	-	1 (3.8)	-	-	-	-
Black	6 (4.8)	-	-	3 (6.7)	3 (10.7)	-	-
Marital status							
Single	41 (32.8)	2 (50.0)	9 (34.7)	15 (33.3)	7 (25.0)	3 (37.5)	5 (35.7)
Married	71 (56.8)	2 (50.0)	14 (53.8)	26 (57.8)	18 (64.3)	4 (50.0)	7 (50.0)
Widowed	2 (1.6)	-	1 (3.8)	1 (2.2)	-	-	-
Separated/ Divorced	11 (8.8)	-	2 (7.7)	3 (6.7)	3 (10.7)	1 (12.5)	2 (14.3)
Schooling							
Elementary School	2 (1.6)	-	-	-	1 (3.6)	-	1 (7.1)
High School	22 (17.6)	-	-	-	20 (71.4)	-	2 (14.3)
Higher Education	22 (17.6)	1 (25.0)	6 (23.1)	5 (11.1)	5 (17.9)	3 (37.5)	2 (14.3)
Graduate Studies	79 (63.2)	3 (75.0)	20 (76.9)	40 (88.9)	2 (7.1)	5 (62.5)	9 (64.3)
Children							
No	65 (52.0)	3 (75.0)	17 (65.3)	19 (42.2)	11 (39.3)	7 (87.5)	8 (57.1)
Yes	60 (48.0)	1 (25.0)	9 (34.7)	26 (57.8)	17 (60.7)	1 (12.5)	6 (42.9)

* Mean (Standard Deviation)

**Table 2 -:** Occupational characteristics of health professionals (n=125). Brazil, 2021-2022

Characteristics	Total (n=125)	Dentists (n=4)	Physical Therapists (n=26)	Nurses (n=45)	Nursing technicians/ Assistants (n=28)	Physicians (n=8)	Others (n=14)
Workplace							
Primary Care	40 (32.0)	4 (100.0)	8 (30.8)	15 (33.3)	6 (21.4)	2 (25.0)	5 (35.7)
Hospital Care	61 (48.8)	-	17 (65.4)	22 (49.0)	13 (46.4)	5 (62.5)	4 (28.7)
Emergency Care	12 (9.6)	-	-	4 (8.9)	6 (21.4)	-	2 (14.3)
Outpatient Care	4 (3.2)	-	-	1 (2.2)	1 (3.6)	1 (12.5)	1 (7.1)
Psychosocial Care	5 (4.0)	-	-	2 (4.4)	2 (7.2)	-	1 (7.1)
Home-based Care	3 (2.4)	-	1 (3.8)	1 (2.2)	-	-	1 (7.1)
Time working							
Less than 6 months	7 (5.6)	-	-	1 (2.2)	4 (14.3)	1 (12.5)	1 (7.1)
Between 6 and 12 months	30 (24.0)	3 (75.0)	8 (30.8)	11 (24.4)	2 (7.2)	3 (37.5)	3 (21.5)
Between 2 and 5 years	42 (33.6)	1 (25.0)	8 (30.8)	13 (28.9)	10 (35.7)	2 (25.0)	8 (57.2)
Between 6 and 10 years	22 (17.6)	-	6 (23.1)	8 (17.8)	5 (17.9)	2 (25.0)	1 (7.1)
More than 10 years	24 (19.2)	-	4 (15.3)	12 (26.7)	7 (24.9)	-	1 (7.1)
Weekly workload (hour)							
Less than 30	6 (4.8)	-	2 (7.7)	-	1 (3.6)	1 (12.5)	2 (14.3)
30	30 (24.0)	-	19 (73.1)	2 (4.4)	4 (14.3)	-	5 (35.7)
36	21 (16.8)	-	1 (3.8)	11 (24.4)	8 (28.5)	-	1 (7.1)
40	60 (48.0)	4 (100.0)	2 (7.7)	30 (66.8)	14 (50.0)	4 (50.0)	6 (42.9)
More than 40	8 (6.4)	-	2 (7.7)	2 (4.4)	1 (3.6)	3 (37.5)	-
Type of contract							
CLT ^*^	60 (48.0)	2 (50.0)	11 (42.3)	25 (55.5)	11 (39.3)	5 (62.5)	6 (42.9)
Public employee	52 (41.6)	1 (25.0)	10 (38.5)	17 (37.8)	15 (53.6)	2 (25.0)	7 (50.0)
Service provider/ Outsourced	13 (10.4)	1 (25.0)	5 (19.2)	3 (6.7)	2 (7.1)	1 (12.5)	1 (7.1)
Income (MW ^†^)							
From more than 1 to 3	25 (20.0)	1 (25.0)	1 (3.8)	4 (8.9)	13 (46.4)	-	6 (42.9)
From more than 3 to 6	49 (39.2)	1 (25.0)	11 (42.3)	18 (40.0)	13 (46.4)	-	6 (42.9)
From more than 6 to 9	22 (17.6)	1 (25.0)	7 (27.0)	12 (26.7)	1 (3.6)	-	1 (7.1)
More than 9	25 (20.0)	1 (25.0)	6 (23.1)	11 (24.4)		7 (87.5)	-
Prefers not to answer	4 (3.2)	-	1 (3.8)	-	1 (3.6)	1 (12.5)	1 (7.1)
More than one employment contract	39 (31.2)	1 (25.0)	13 (50.0)	13 (28.9)	6 (21.4)	2 (25.0)	4 (28.7)

* CLT = *Consolidação das Leis do Trabalho* (Consolidation of Labor Laws);

† MW = Minimum Wage in force in 2022 (R$ 1,212.00, equivalent to US$ 249.38)

A large part of the professionals (55%) reported practicing physical activity; however, 59.2% had Body Mass Index (BMI) values greater than 25 (overweight and obesity). Forty-two percent of the participants reported having some disease; however, a higher number reported using medications (66%). Tobacco use was mentioned by 11% of the participants and 58% reported never drinking alcohol or doing so once or fewer times a month. Most of the participants did not present depression symptoms (54%); however, the burnout and stress risks in a large part of the sample (86% and 81%, respectively) drew the attention. Exposure to at least one type of offensive behavior was reported by forty-four percent of the participants, with emphasis on nursing technicians/assistants (54%) and nurses (51%). Table 3 presents these and other data from the participants’ health profile, according to professional category. 

**Table 3 -:** Health characteristics of health professionals (n=125) and the occurrence of depressive symptoms and offensive behaviors at work. Brazil, 2021-2022

Characteristics	Total (n=125)	Dentists (n=4)	Physical Therapists (n=26)	Nurses (n=45)	Nursing technicians/ Assistants (n=28)	Physicians (n=8)	Others (n=14)
Physical activity	69 (55.2)	3 (75.0)	16 (61.5)	24 (53.3)	10 (35.7)	8 (100.0)	8 (57.1)
BMI ^*^ (kg/m ^2^)	27 (5.9)^†^	25.0 (5.3)^†^	27.5 (7.5)^†^	27.3 (4.8)^†^	28.8 (6.7)^†^	25.2 (2.3)^†^	24.4 (4.6)^†^
Underweight	1 (0.8)	-	1 (3.8)	-	-	-	-
Normal weight	50 (40.0)	3 (75.0)	9 (34.6)	18 (40.0)	9 (32.1)	2 (25.0)	9 (64.3)
Overweight	41 (32.8)	-	8 (30.8)	12 (26.7)	11 (39.3)	6 (75.0)	4 (28.6)
Obesity	33 (26.4)	1 (25.0)	8 (30.8)	15 (33.3)	8 (28.6)	-	1 (7.1)
Mentions some disease	53 (42.4)	-	9 (34.6)	19 (42.2)	17 (60.7)	5 (62.5)	3 (21.4)
Uses medication	83 (66.4)	3 (75.0)	14 (53.8)	28 (62.2)	21 (75.0)	8 (100.0)	9 (64.3)
Smoking	14 (11.2)	-	2 (7.7)	3 (6.7)	5 (17.9)	-	4 (28.6)
Alcohol consumption							
Never	37 (29.6)	1 (25.0)	7 (26.9)	18 (40.0)	8 (28.6)	1 (12.5)	2 (14.3)
1 time/month or less	35 (28.0)	1 (25.0)	6 (23.1)	13 (28.9)	9 (32.1)	-	6 (42.9)
2 - 3 times/month	33 (26.4)	-	9 (34.6)	9 (20.0)	6 (21.4)	4 (50.0)	5 (35.7)
2 - 3 times/week or more	20 (16.0)	2 (50.0)	4 (15.4)	5 (11.1)	5 (17.9)	3 (37.5)	1 (7.1)
Depression (BDI-II ^‡^)	13.0 (5.0-18.0) ^‡^	11.5 (6.5-27.0) ^‡^	9.0 (2.0-14.8) ^‡^	13.0 (5.0-21.0) ^‡^	14.0 (10.0-21.0) ^‡^	12.5 (3.0-15.8) ^‡^	12.5 (2.8-16.3) ^‡^
No depression	68 (54.4)	3 (75.0)	16 (61.5)	24 (53.3)	13 (46.4)	5 (62.5)	7 (50.0)
Average depression	27 (21.6)	-	6 (23.1)	7 (15.6)	6 (21.4)	2 (25.0)	6 (42.9)
Moderate depression	20 (16.0)	-	4 (15.4)	9 (20.0)	5 (17.9)	1 (12.5)	1 (7.1)
Severe depression	10 (8.0)	1 (25.0)	-	5 (11.1)	4 (14.3)	-	-
Burnout risk	107 (85.6)	3 (75.0)	21 (80.8)	40 (88.9)	24 (85.7)	7 (87.5)	12 (85.7)
Stress risk	101 (80.8)	3 (75.0)	19 (73.0)	38 (84.4)	21 (75.0)	7 (87.5)	13 (92.9)
Offensive behaviors	55 (44.0)	2 (50.0)	7 (26.9)	23 (51.1)	15 (53.6)	4 (50.0)	4 (28.6)

* BMI = Body Mass Index;

† Mean (Standard Deviation);

‡ BDI-II = Beck Depression Inventory II;

§ Median (p25-p75)

Of the total number of participants, 55 (44%) reported one or more types of offensive behavior, totaling 83 episodes that characterize violence at work (some participants reported more than one type of behavior, as shown in Figure 1). Threats of violence were the most frequent offensive behavior, reported by 32 professionals (26%) and more frequent among nurses (33%; n=15), nursing technicians/assistants (36%; n=10) and physicians (38%; n=3). Unwanted sexual attention was reported by 19 participants (15%), being more frequent in dentists (25%; n=1) and nurses (24%; n=11). Physical violence affected 11 professionals (8.8%) and was more reported by nursing technicians/assistants (21%; n=6). As for bullying, 21 professionals (17%) were victims of this type of offensive behavior, especially dentists (25%; n=1) and nursing technicians/assistants (21%; n=6). 

Table 4 presents the profile of the aggressors in relation to the type of offensive behavior reported by the participants. Most of the offensive behaviors were practiced by patients, with the exception of bullying, perpetrated by co-workers (48%) and supervisors (26%). 

The analysis of the association between the dimensions from Domain 7 - “Offensive behaviors” presented non-significant results (P>0.05) in all comparisons, which means that the occurrence of a given offensive behavior is not associated with the occurrence of others.

**Figure:**
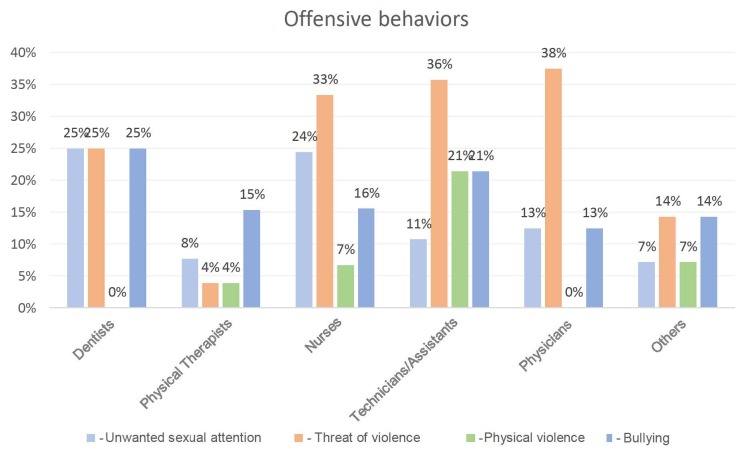
Figure 1- Distribution of the health professionals (n=125) exposed to unwanted sexual attention, threats of violence, physical violence and bullying. Brazil, 2021-2022

**Table 4 -:** Distribution of the profile of aggressors in relation to the type of offensive behavior committed to health professionals. Brazil, 2021-2022

Offensive behaviors	Patients	Co-workers	Supervisors	Subordinates
Unwanted sexual attention	59.1%	27.3%	13.6%	-
Threat of violence	75.7%	13.5%	5.4%	5.4%
Physical violence	100%	-	-	-
Bullying	18.5%	48.2%	25.9%	7.4%

There was no association between threats of violence and burnout risk ( *P*>0.05) or between threats of violence and stress risk ( *P*>0.05). There was also no significant association between violence at work and gender ( *p*=0.32), that is, this phenomenon is independent of sex. The logistic regression analysis partially confirmed this finding; in other words, that offensive behaviors were not associated with female gender (OR: 1.34; 95% CI: 0.51-3.50; *p*=0.55) and with having children (OR: 0.64; 95% CI: 0.31-1.31; *p*=0.22), and that they were also not associated with stress (OR: 2.20; 95% CI: 0.84-5.76; *p*=0.11). On the other hand, the regression indicated a significant association with burnout (OR: 4.73; 95% CI: 1.29-17.3; *p*=0.02) and with depression symptoms (OR: 1.05; 95% CI: 1.01-1.10; *p*=0.02). Thus, burnout increased by 4.73 times the chances of workers suffering offensive behaviors at work and presence of depressive symptoms increased by 1.05 times these chances. 

## Discussion

The occurrence of offensive behaviors, that is, of different forms of violence at work, directed at SUS health workers was high, similarly to what has been pointed out in international research studies ^(^ 3 ^,^ 4 ^,^ 5 ^-^ 6 ^,^ 25 ^)^, which reinforces the urgency of addressing this problem and intervening in this context. 

A meta-analysis with 78 articles from different regions of the world, encompassing 39,898 participants, estimated 62% prevalence of violence at work in health, practiced by patients and visitors. Non-physical violence had 43% prevalence, with verbal abuse as the most common form (58%), followed by threats of violence (33%) and sexual harassment (12%); physical violence had 24% prevalence ^(^ 5 ^)^. The findings of the current research also point to higher prevalence of non-physical violence, such as threats of violence (26%) and unwanted sexual attention (15%); physical violence was responsible for 8.8% of the cases. 

The prevalence of bullying (17%) in this study was similar to the one found in a study carried out with 2,810 health professionals (from 4% to 18% ^(^ 26 ^)^) and slightly lower than the results of a literature review that included 28 international studies (mean prevalence of 26%) and indicated higher prevalence values in physicians (32%) and nurses (31%) ^(^ 27 ^)^, differing from this study, which found higher prevalence in dentists (25%) and nursing technicians/assistants (21%). 

Other research studies, with sample sizes similar to this one ^(^ 28 ^-^ 29 ^)^ and higher ^(^ 6 ^,^ 30 ^)^ and which evaluated different types of violence at work, showed high prevalence against nursing workers, the most exposed category when compared to other professionals in the health area ^(^ 6 ^)^. The findings of this research also point to Nursing workers, especially technicians and assistants, as the most vulnerable victims to different types of violence. 

Some factors explain the higher occurrence of violence in Nursing, such as direct and frequent contact with patients ^(^ 31 ^)^, the aggressors’ anguish or suffering ^(^ 32 ^)^, the stressful context ^(^ 32 ^)^, precarious working conditions and the very development of the profession in the historical-social context ^(^ 33 ^)^. Providing direct assistance to users in different health services and interacting with other team members favor access to these workers in times of complaints and conflicts ^(^ 15 ^)^, turning them into easy targets for abusive behaviors. 

Despite not being a finding of this and another study ^(^ 5 ^)^, Nursing is strongly related to situations of physical, psychological and sexual violence because it is a predominantly female profession ^(^ 34 ^)^, with studies that point to the implication of gender in situations of violence, showing that women are more exposed than men ^(^ 2 ^,^ 6 ^)^. 

A literature review pointed out that verbal violence against physicians at work is more prevalent than physical violence ^(^ 35 ^)^, corroborating the findings of this research. Violence against physicians is mainly influenced by factors related to patient dissatisfaction and low impulse control, as well as by organizational factors such as infrastructure problems, communication failure and poor management ^(^ 35 ^)^. 

As is the case with the findings of this research, most of the articles mention patients as the main aggressors ^(^ 6 ^,^ 16 ^,^ 30 ^-^ 31 ^,^ 36 ^)^ in different types of violence, with the exception of bullying, which by its very design ends up being perpetrated by co-workers, followed by superiors ^(^ 26 ^)^. 

Violence perpetrated by patients stems from the profile of the aggressors, such as characteristics of the pathologies, gender, age, history of violence, neurological disorders, history of alcohol/drug abuse, mental disorders and social and economic status, in addition to the precarious conditions of the health services, which may result in lower care quality and even deterioration of the patients’ conditions due to missed care and/or lack of resources available for treatment ^(^ 6 ^,^ 14 ^-^ 15 ^)^. 

Such factors, especially those related to the patients’ pathologies, are mentioned by the workers themselves to rationally justify situations of violence and redeem the patients’ guilt ^(^ 6 ^,^ 14 ^)^. This thinking contributes to the normalization of violence at work in health, discouraging its notification by workers and rendering the critical situation invisible, as well as the necessary confrontation by managers ^(^ 15 ^)^. 

Violence at work affects the professionals’ quality of life and well-being, contributing to the increase in the levels of anxiety, anger, depression and feeling of guilt ^(^ 4 ^)^. Violence at work can have consequences for health institutions themselves due to absenteeism resulting from work-related accidents or diseases related to violence, burnout and decreased job satisfaction, which exerts impacts on care quality, budgets and increased costs for the institutions ^(^ 4 ^)^. 

A significant number of participants were at risk of burnout (86%) and stress (81%) in this research and burnout increased by almost five times the chances of workers suffering offensive behaviors. A literature review also found an association between exposure to bullying and burnout, depression, psychological stress and anxiety, among other mental health problems ^(^ 27 ^)^. On the other hand, a literature review identified an inverse relationship, associating violence at work with higher incidence values of burnout, lower job satisfaction, lower patient safety, depression, anxiety and other adverse consequences ^(^ 4 ^)^. 

A research study conducted with 539 mental health nurses observed the presence of high levels of occupational stress and exposure to violence at work, which suggests that violence at work can also contribute to aggravating the occupational stress level, especially in the long term ^(^ 37 ^)^. 

This research found an association between the occurrence of offensive behaviors and depression symptoms, a finding also detected in a literature review ^(^ 38 ^)^ and in a Chinese study conducted with 3,426 health professionals which, in addition, observed that being a nurse and having a disease are risk factors associated with depression ^(^ 3 ^)^. 

Not only violence, but also other psychosocial risk factors are associated with the burnout and depression risks among health professionals, such as exhausting hour loads, lack of material resources, fear of being infected and of infecting others, exposure to large-scale deaths and sleep impairments ^(^ 39 ^)^, aspects intensified in the first two years of the COVID-19 pandemic, the data collection context. In turn, the risks of burnout (which is a result of occupational stress) and of depression contribute to an increase in violence at work, generating a cyclical movement that needs to be interrupted. 

This study has some limitations. Despite the countless recruitment strategies throughout the national territory, the small sample size and low geographic representation do not support generalizations for a continental and diversified country such as Brazil. The possibility of selection bias is acknowledged, as disclosure was made through digital media and data collection was electronic, restricting access and participation of part of the target population (there was no treatment of possible biases).

Despite the limitations, the findings make it possible to reflect on the impact of violence on workers’ health, contributing knowledge about the characteristics related to violence in different professional categories and its association with burnout and depression, important risk factors that need attention, especially in view of the consequences brought about by the COVID-19 pandemic, which accentuated already existing weaknesses in health services. These findings can encourage not only the notification and report of situations of violence against health professionals, but also boost the development of actions aimed at combating and preventing these situations by managers, mainly considering the risk factors highlighted, the profile of the aggressors and the particularities of each professional category, in order to avoid the growing increase in violence at work, as well as to promote actions aimed at welcoming victims of aggression, including psychological and organizational support.

## Conclusion

The occurrence of offensive behaviors directed at SUS workers is frequent and characteristic, with threats of violence as the most common type of behavior and, therefore, it constitutes a priority problem in the planning of measures for its confrontation and prevention.

The findings point to the relevance of understanding the psychosocial aspects related to work organization that favor greater exposure of the Nursing team and the aggressions mainly perpetrated by patients, in order to map effective strategies against violence at work in health. It is urgent to adopt measures that inhibit offensive practices among co-workers and at different hierarchical levels, in order to build a healthy and collaborative work environment.

The association between the occurrence of offensive behaviors, burnout and depressive symptoms indicates the need for greater attention to workers’ mental health, especially considering the consequences generated and aggravated by the COVID-19 pandemic context, with the intention of also reducing occupational violence. In future research studies, it is recommended to evaluate instruments developed for preventing and combating violence related to work in health.
